# Developmental progression of intellectual disability, autism, and epilepsy in a child with an IQSEC2 gene mutation

**DOI:** 10.1002/ccr3.1139

**Published:** 2017-08-24

**Authors:** Rachelle Zipper, Sherri D. Baine, Jacob Genizi, Hen Maoz, Nina S. Levy, Andrew P. Levy

**Affiliations:** ^1^ Technion Faculty of Medicine Technion Israel Institute of Technology Haifa Israel; ^2^ Department of Pediatrics North York General Hospital University of Toronto Toronto Ontario Canada; ^3^ Pediatric Neurology Unit Bnai Zion Medical Center Haifa Israel; ^4^ Kiryat Shmona Regional Unit for Child Development Kiryat Shmona Israel

**Keywords:** Autism, child development, epilepsy, intellectual disability, IQSEC2

## Abstract

The neurodevelopmental progression of a school‐aged child with a spontaneous IQSEC2 mutation has demonstrated apparent regression of milestones and language. Seizures associated with the disorder have been refractory to medical treatment. Late treatment of autism in this child has led to improved social skills.

## Introduction

### IQSEC2

*IQSEC2* is an X‐linked gene whose protein product is localized on excitatory synapses as part of the NMDA receptor complex, and has been proposed to play a role in α‐amino‐3‐hydroxy‐5‐methyl‐4‐isoxazolepropionic acid (AMPA) receptor trafficking, and thus in synaptic plasticity 1. Clinically, mutations in *IQSEC2* have been associated with a syndrome of autism 2, 3, intellectual disability, and epilepsy 4. *IQSEC2* mutations appear to account for approximately 10% of patients with intellectual disability and seizures referred for diagnostic exome sequencing 5. A detailed description of the developmental progression of disabilities associated with IQSEC2 mutations has not been described. Attempted treatments to alleviate some of the disabilities associated with the disease have not been documented. In this case report, we describe the neurodevelopmental profile of a child with a spontaneous IQSEC2 mutation over the course of his first 5 years. His disease and its refractory nature to treatment, coupled with the apparent regression of milestones and language, will help to better define the natural history of this genetic disorder and serve as a necessary step in assessing the efficacy of new treatments that can show real clinical benefit.

## Case Report

The subject of this report is E.H., a 5‐year‐old male child who has a spontaneous mutation in the IQ domain of *IQSEC2* (A350V) identified by whole‐exome sequencing. EH was born full term, following a normal pregnancy to nonconsanguineous parents. Developmental milestones were achieved at the appropriate time through the first 8 months of life. Suddenly at 8 months of age, EH began to clinically manifest this mutation with the onset of seizures and severe developmental regression. Attempts at treatment with a chronological comparison of epileptic, behavioral, and motor development until the present (age 5) are provided in Table 1.

**Table 1:** Neurodevelopmental progression in EH

Age	Seizures	Socialization	Language	Repetitive behaviors	Motor development	Treatment
6 months	None	Smiles responsively	Vocalizing	None	Normal tone	
8–12 months	Upward deviation eyes and head drop lasting 5 sec every 2 min; confirmed by video EEG as partial complex seizure	No recognition or interaction with parents	Regression and loss of vocalizing	Flicking tag of toys/pillow	Delayed –failing to sit and crawl	Oxcarbazepine increased seizure; Levetiracetam
12–18 months	No change in seizure frequency or duration	Does not seek comfort or recognize parents	No vocalization	Flicking tag and stroking soft fabrics	Little movement	Physical therapy initiated;
18–24 months	No change in seizure frequency or duration		Speaks three words, uses sign gestures to communicate		Reaching milestones crawling and walking with irregular gait by age 2	Levetiracetam
2–2.5 years	Onset of drop attacks and Salaam movement occurring every 5–6 min		Loss of vocalization and use of signing	Flicking; repetitive play with same toy	Decrease in activity but still walking	Successively valproate; topiramate; vigabatrin, ACTH; ketogenic diet and clobazam
2.5–3 years	Tonic clonic seizure all limbs, falling down; drooping of head; 1 min duration no postictal state 5–6 times per hour	Does not seek interaction with parents; does not respond to his name; does not respond to No	No vocalizations Hums some songs	Opening and closing Velcro and cupboard doors	Walks independently; runs and climbs and throws objects with both hands; able to feed himself	Special education nursery initiated
3–3.5 years	Tonic clonic seizure 2–3 per hour		No vocalizations Hums songs	Hypersensitivity to sounds and covers ears with loud noises		All medications stopped due to allergic reaction
3.5–4 years	Prolonged tonic clonic seizures; seizure‐free periods of 3 weeks followed by 2–3 days with 3–4 tonic clonic seizure with sleeping 2 h postictal state	Brings parents hand to object he wants; does not seek parental comfort and no reciprocal play	No vocalizations	Plays with Legos repeatedly putting together and taking apart; buckling and unbuckling snaps		Long (5′) seizures terminated with midazolam; chronic valproate and rufinamide
4–5 years	Tonic clonic seizure 6–8 times month 1–2 min duration	Seeking interactions children/parents; independent play	Hums songs no recognizable words	Decreased repetitive requests for object	Gross and fine motor skills normative for age	Intensive treatment autism; cannabidiol added to valproate and rufinamide

### Epilepsy

At 8 months of age, episodes in which EH had an upward deviation of the eyes every 5 min that lasted several seconds were noted. Video EEG confirmed partial complex seizures. Table 1 provides chronological changes in seizure pattern, duration, and frequency as well as treatments attempted.

### Motor development

No specific delays in gross or fine motor development were noted in the first 6–8 months of life. Coinciding with the onset of seizures, it was observed that motor milestones had plateaued, and at 8 months, the child was not sitting independently or crawling. Physiotherapy initiated at 8 months appeared to facilitate his achieving motor milestones so that by 24 months of age, he was capable of walking with an irregular gait and no support. At age 5, EH can walk, run, climb, and throw objects with both hands. Regarding his fine motor skills, EH can feed himself with utensils and manipulate small objects.

### Autism

#### Social communication and social interaction summary

At 4 years of age, EH would bring his parents’ hand to a desired object in order to communicate a need such as wanting a drink or snack. EH did not respond to his name, nor to the commands “no!” or “look!” The child was unable to identify body parts or common objects in a book such as a car, train, or dog. EH seemed very content to be on his own. He did not seek parental comfort and demonstrated no interest in children to the point of walking over them or bumping into them as if they were not there. The child did not demonstrate reciprocal play such as back‐and‐forth rolling or throwing a ball with parents.

#### Language impairment summary

EH has shown significant regression in his language skills. Early vocalizations present at 6 months of age were absent at 9 months of age. Subsequent speech development has been irregular, with periods of increased vocalization followed by regression. At 18 months of age, EH had the ability to say several words such as “amen”, “all done,” and “thank you”; however, this ability disappeared by 2 years of age. EH was taught sign language at 2 years of age, allowing him to express his desire for water, food, or a bath. These skills also disappeared by 3 years of age. At age 5, EH can make various noises but no obvious word approximations despite intensive speech therapy.

#### Restrictive and repetitive behavior summary

From 12 months of age, the child was noted to enjoy engaging in flicking tags. At 3 years of age, he began to further demonstrate repetitive activities such as clasping and unclasping buckles and buttons, assembling and disassembling two pieces of Lego, and opening and closing Velcro and cupboard doors. He also enjoyed bubbles, stroking soft fabrics, flicking tags or tickets on clothing, and flapping and watching his hands.

#### Altered response to sensory input

From 12 months of age, EH was noted to have apparent hyporeactivity to pain. For example, the child did not cry during a lumbar puncture at 12 months of age or during a full level skin biopsy performed at age 2 with only local anesthetic. EH demonstrates hypersensitivity to certain sounds, which caused him to get upset such as the shower and dripping water. The child will often cover his ears in response to loud noises.

### Diagnosis of autism

At age 4, a formal diagnosis of autism spectrum disorder was made based on the Autism Diagnostic Observation Schedule (ADOS‐2), Childhood Autism Rating Scale (CARS‐2) questionnaire, and Diagnostic and Statistical Manual of Mental Disorders (DSM‐5) criteria 6. While this diagnosis was not formally made until the child was 4 years old, there were many earlier clues in the child's social interactions, behavior, and communication skills that suggest that he manifested autism at a significantly earlier age, as described above. Factors such as his uncontrolled epilepsy, intellectual disability, and the presence of apparent eye contact with anyone from a young age may have contributed to the delay in making the diagnosis of autism spectrum disorder (ASD) at an earlier age.

### Intellectual disability

EH's diagnosis of ASD is comorbid with intellectual disability. The Stanford–Binet Intelligence Scale 5 for early childhood at age 4 showed that EH had low levels of functioning (FSIQ = 44), scoring higher on the nonverbal scale (NVIQ = 48) than the verbal scale (VIQ = 46). He showed most interest and motivation in the subtest that required matching shapes to a board.

### Treatment and outcomes

#### Epilepsy

EH has received many antiseizure medications and a trial of the ketogenic diet. The current regimen of valproic acid, cannabidiol, and rufinamide has resulted in the longest seizure‐free periods. However, complete remission of seizures has not been achieved.

#### Special education

EH began attending a specialized program for children with developmental disabilities at age 2. At this learning center, he received sensory, occupational, and speech therapy but demonstrated no progress in learning new skills. After receiving a diagnosis of ASD at age 4, he began twice‐weekly treatment of 6 h sessions at a center dedicated for autism using integrated approaches including floor time and applied behavior analysis (ABA). Since the start of his treatment for autism, he has demonstrated a greater effort to make contact with others. EH is now approaching all persons in a room and touching them gently on the arm while making eye contact. He is trying new activities such as playing with a new toy or allowing himself to be dressed up in a costume and smiling while in the costume. While in the past after being given a pen he would just take the cap on and off, he will now take the pen and draw with it or play hide and seek with it. He will also now play hide and seek with his parents and laugh during activities that he does with them. He seems to be less frustrated by not being able to get what he wants.

## Discussion and Outlook

IQSEC2 mutations have been characterized in several children with early onset epilepsy and intellectual disability with considerable variability in their severity. Notably, the age of onset of EH's case at 8 months appears to be the earliest of those reported in the literature, compared to previously reported cases that generally state onset of the disease at age 4.

The apparent regression in language skills exhibited by EH may have been a result of his severe seizure disorder 7. However, other areas of regression in social communication and the development of repetitive behaviors were a major clue to ASD 8. Children whose social skills decline (such as responsive smile, joint referencing, looking to the parent for reassurance, and shared interest) should be evaluated for autism 9. Furthermore, repetitive/restrictive behaviors, using the parents hand as a tool, and hyporeactivity to pain should be considered red flags for autism. Diagnosing autism in a child with MID is challenging but the key is usually a delay in social skills that is out of proportion to other developmental skills. In the case of EH, he is able to scroll through an iPhone and find a favorite song but does not inhibit to “No” or respond to his name, a skill one would typically expect in a 12‐month‐old child.

Best practice for ASD involves early diagnosis and treatment in the form of intensive behavior therapy 10, 11. As more children with MID and autism are identified, it will be important to assess whether early intervention for autism is most helpful earlier in the child's chronological age or whether it is still as helpful in a child who may be chronologically older but cognitively functions at a lower level. ASD treatments are costly and time‐consuming and worth it. Here, we report on a child with severe seizures, intellectual disability and ASD who was diagnosed at four and is still able to learn new skills. Clinicians can offer parents hope of improved quality of life even in children with severe impairments.

## Approval and Consent

The parents of EH have provided informed consent to use his medical record and information in this case report.

## Authorship

RZ: prepared and edited manuscript and helped with literature review. SB: prepared and edited the manuscript and helped with literature review. NSL: prepared and critically edited the manuscript. JG: edited the manuscript and provided clinical data on the patient. HM: edited the manuscript and provided clinical data on the patient. APL: wrote the manuscript and collated comments of all authors into its final form.

## Conflict of Interest

None declared.
